# Recurrent Abdominal Pain Caused by a Parasitic Leiomyoma Containing Endometrial Cells Following Laparoscopic Hysterectomy

**DOI:** 10.7759/cureus.84391

**Published:** 2025-05-19

**Authors:** Ai Kusakabe, Hiroshi Tsubamoto, Naohito Beppu, Tomoko Ueda, Seiji Mabuchi

**Affiliations:** 1 Department of Obstetrics and Gynecology, School of Medicine, Hyogo Medical University, Nishinomiya, JPN; 2 Department of Surgery, School of Medicine, Hyogo Medical University, Nishinomiya, JPN

**Keywords:** abdominal pain, endometrial cells, laparoscopic hysterectomy, manual morcellation, parasitic leiomyoma

## Abstract

Parasitic leiomyomas are rare complications of uterine morcellation after laparoscopic hysterectomy (LH). These tumors typically consist of smooth muscle cells and are often asymptomatic. However, parasitic leiomyomas containing endometrial cells that exhibit periodic abdominal pain have not been reported previously. A 47-year-old woman presented with recurrent right lower abdominal pain two years after undergoing LH and bilateral salpingectomy for uterine leiomyoma. A contrast-enhanced computed tomography revealed a 2.5-cm mass lesion between the lateral aspect of the ascending colon and the abdominal wall. Surgical excision revealed that the mass was firmly adherent to the abdominal wall and the omentum. Histopathological examination confirmed the diagnosis of iatrogenic parasitic leiomyoma with endometrial cells composed of endometrial glands and endometrial stroma, smooth muscle proliferation, and internal hemorrhage. It was concluded that her periodic abdominal pain was caused by the menstrual-like bleeding within a parasitic leiomyoma. Postoperative recovery was uneventful, with symptom resolution. Follow-up imaging and clinical evaluations showed no recurrence.

## Introduction

During laparoscopic hysterectomy (LH), large fibroids must be morcellated and extracted either via the transvaginal route or through a small abdominal incision to remove the tissue from the abdominal cavity. Residual tissue fragments left in the abdominal cavity during morcellation can result in parasitic leiomyomas, which may receive blood from surrounding organs [1,2].

A definitive diagnosis of parasitic leiomyoma is established through surgical tissue removal and subsequent pathological evaluation. Most cases of parasitic leiomyomas are histologically composed entirely of smooth muscle cells [2]. However, there have been no reports of parasitic leiomyomas containing endometrial cells that exhibit hormone-dependent, menstrual-like bleeding.

The clinical symptoms of parasitic leiomyomas depend on their location and size. They are often asymptomatic and incidentally discovered during examinations or surgeries [3]. Cases of parasitic leiomyomas presenting with abdominal pain have been reported, with mechanisms such as tumor degeneration [4], traction pain on the round ligament due to tumor enlargement [5], and torsion [6]. However, there have been no reports of pain attributed to bleeding from a parasitic leiomyoma containing endometrial cells. Here, we present a case of parasitic leiomyoma with hormone-dependent, menstrual-like bleeding originating in the smooth muscle containing endometrial cells, which was identified following recurrent lower abdominal pain after LH.

This article was presented as a poster at the 76th Annual Meeting of the Japan Society of Obstetrics and Gynecology on April 19, 2024.

## Case presentation

**Figure 1:**
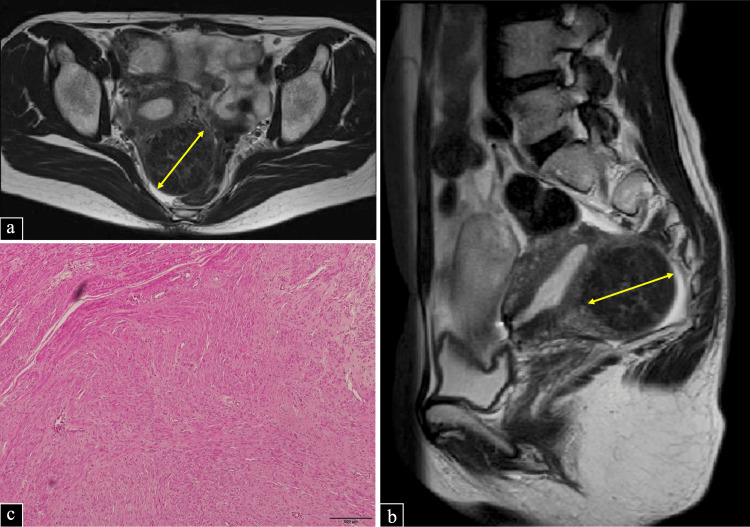
MR and histopathological images of previous total laparoscopic hysterectomy. (a,b) T2-weighted MR images showing multiple uterine fibroids measuring up to 56 mm. (c) Histopathological examination of the excised uterine myoma revealing proliferation of smooth muscle cells MR: magnetic resonance

A 47-year-old woman, para three (three cesarean sections), underwent LH and bilateral salpingectomy for uterine leiomyoma (5.6 × 4.2 cm in size), as shown in Figures 1a, 1b, with hypermenorrhea and dysmenorrhea. Since the patient had no history of vaginal delivery, the umbilical wound was extended, and the resected uterus was removed from the abdominal cavity by manual morcellation. The resected uterus was guided under a 5-cm skin incision extending the umbilical incision. The uterovaginal area was transabdominally grasped and pulled with single or double hook forceps. In contrast, a conical or tubular incision was made from the inside using a scalpel or scissors, and the weight was reduced from the inside and finally removed through the umbilical incision. So-called “in-bag morcellation” was not performed during uterus retrieval. Histopathological examination revealed uterine leiomyoma (Figure 1c) with no evidence of adenomyosis or leiomyosarcoma.

Approximately two years after the surgery, the patient experienced right lower abdominal pain, which resolved spontaneously within three to four days. Over the next year and a half, the same symptoms recurred every six months. Eventually, the pain became so severe that she had to be hospitalized. Blood test results showed a mild inflammatory reaction. Tumor markers were carbohydrate antigen-19-9 at 5.6 U/mL and carcinoembryonic antigen at 2.4 ng/mL, neither of which was elevated.

**Figure 2:**
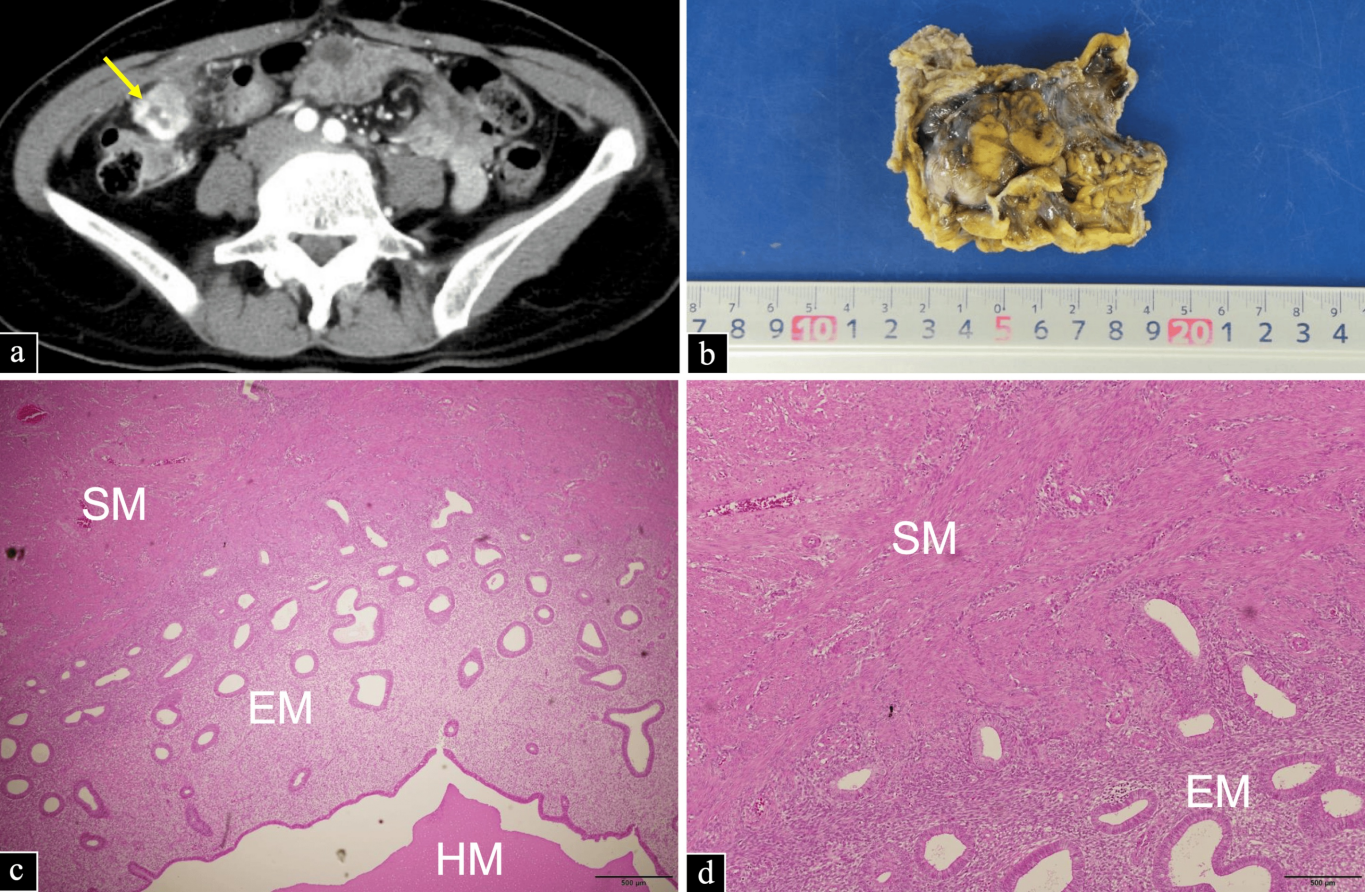
Preoperative images, explanted specimen, and histopathological images of parasitic leiomyoma. (a) The contrast-enhanced computed tomography image shows a mass lesion measuring 2.5 cm in diameter located just beneath the abdominal wall in the right lower quadrant (yellow arrow). (b) Excised specimen showing a relatively well-defined grayish-white solid mass with intratumoral hemorrhage. Pathological images of the excised specimen: (c) bundles of SM-like cells developed above the image, and EM cells consisting of endometrial glands and endometrial stroma are present below the center of the image. HM within the EM cells is observed in the lower part of the image, and (d) smooth muscle-like cells are observed in the upper part of the image, and EM cells are observed in the lower part EM: endometrioid; HM: hemorrhage; SM: smooth muscle

A contrast-enhanced CT scan revealed a mass lesion approximately 2.5 cm in diameter with a low-density area in the ascending colon, correlating with the site of pain (Figure 2a). Differential diagnosis included submucosal tumors of the colon, colorectal cancer, and abscess formation. Lower gastrointestinal endoscopy was performed, but no lesions were detected in the intestinal tract. Consequently, the decision was made to surgically remove the tumor for both diagnostic and therapeutic purposes.

The procedure was performed under laparoscopic assistance, using a 5-cm midline longitudinal incision in the umbilical region, a 12-mm incision in the left side of the abdomen, and a 5-mm incision in the left lower abdomen. During surgery, intra-abdominal observations revealed that the mass originated from the omentum and was partially and firmly adherent to the abdominal wall. A portion of the mass adhering to the abdominal wall was dissected from the internal abdominal oblique muscle, and the mass was excised en bloc along with the omentum to ensure a sufficient surgical margin (Figure 2b). The bilateral ovaries were normal in appearance, and there were no obvious peritoneal nodules, including in the pelvis. Therefore, it was decided to perform surgery only to remove the tumor for diagnostic purposes.

Histopathological examination demonstrated that the tissue was rich in intervening endometrial cells, comprising glandular tissue and stroma, along with smooth muscle cell proliferation and internal hemorrhage (Figures 2c, 2d). Based on these findings, iatrogenic parasitic uterine leiomyoma was diagnosed after LH surgery. No additional lesions were identified in the abdominal cavity, and the patient’s symptoms improved postoperatively.

Approximately one year later, during follow-up, E2 levels declined, marking the onset of menopause. At the three-year follow-up, the patient remained asymptomatic, and a CT scan showed no evidence of recurrence.

Written informed consent was obtained from the patient for using patient records and any accompanying images.

## Discussion

We experienced a very rare case of parasitic leiomyoma with hormone-dependent, menstrual-like bleeding originating in the smooth muscle containing endometrial cells, which was identified following recurrent lower abdominal pain after LH.

This case report highlights two novel clinical findings. First, a parasitic leiomyoma containing endometrial cells exhibiting hormone-dependent, menstrual-like bleeding within the identified mass. Second, periodic abdominal pain caused by bleeding from such a parasitic leiomyoma can trigger its detection.

Most parasitic leiomyomas are histologically composed entirely of smooth muscle cells [2]. One case has been reported in which the histology of a parasitic leiomyoma was diagnosed as adenomyosis following surgery for uterine adenomyosis [7]. However, no reports have described parasitic leiomyomas containing endometrial cells and exhibiting hormone-dependent, menstrual-like bleeding in the mass.

The mechanism underlying iatrogenic parasitic leiomyomas involves the implantation of shredded uterine tissue into the pelvis, where it receives blood flow through neovascularization from the omentum and peritoneum [8-10]. In our case, the parasitic leiomyoma, which contained endometrial cells and received blood flow from neovascular vessels, was influenced by female hormones of the remaining ovaries, leading to menstrual-like bleeding in the mass.

 Parasitic myomas may be found asymptomatically and incidentally [10-12] or may present with abdominal symptoms. Asymptomatic parasitic leiomyomas were identified in 13.8% of cases during a second operation following laparoscopic myomectomy without in-bag morcellation [13].

Abdominal pain is a key symptom leading to the identification of a parasitic leiomyoma. Abdominal pain associated with parasitic leiomyomas has previously been attributed to tumor degeneration [4], traction on the round ligament due to tumor enlargement [5], and torsion [6]. As there was no evidence of degeneration or torsion, the pain in our case was considered to result from hormone-dependent bleeding with parasitic leiomyoma.

A notable feature of the abdominal pain in this case was its cyclic nature, which occurred every six months. To the best of our knowledge, no previous report has described parasitic leiomyomas presenting with periodic abdominal pain. It remains unclear whether the six-month intervals were due to proximity to menopause or the small volume of blood loss with each episode. Periodic abdominal pain may serve as a clinical indicator for the detection of parasitic leiomyomas presenting with hormone-dependent, menstrual-like bleeding.

The U.S. FDA recommends using containment systems during power morcellation in laparoscopic procedures for uterine fibroids to prevent unexpected intraperitoneal dissemination of uterine sarcomas [14]. Containment bag systems can also prevent the formation of parasitic leiomyomas. Currently, there are no strong recommendations for using containment bag systems during manual morcellation of large fibroids removed via the transvaginal route or through small abdominal incisions during LH. However, the lesson learned from our case is that when the uterus is shredded and removed via manual morcellation outside the abdominal cavity during LH, the use of containment bag systems is recommended to prevent the occurrence of parasitic leiomyomas.

## Conclusions

We experienced a very rare case of parasitic leiomyoma with hormone-dependent, menstrual-like bleeding originating in the smooth muscle containing endometrial cells. Although very rare, this condition should be kept in mind when women experience periodic abdominal pain after LH for uterine myoma, especially when uterus had been removed from the abdominal cavity or through the vaginal route in case of vaginal hysterectomy by morcellation.
